# Iron (oxyhydr)oxides are responsible for the stabilization of Cu and Zn in AMD after treatment with limestone

**DOI:** 10.7717/peerj.14663

**Published:** 2023-01-30

**Authors:** Yuan Ding, Yan Long, Weiya Wang, Zhe Wei, Shuo Cai

**Affiliations:** 1 National-Local Joint Engineering Research Center of Heavy Metals Pollutants Control and Resource Utilization, Nanchang Hangkong University, Nanchang, China; 2 College of Environment and Chemical Engineering, Nanchang Hangkong University, Nanchang, China; 3 Jiangxi Key Laboratory of Agricultural Efficient Water-Saving and Non-Point Source Pollution Preventing, Jiangxi Central Station of Irrigation Experiment, Nanchang, China

**Keywords:** AMD, Neutralization, Iron (oxyhydr)oxide, Heavy metals, Stabilization mechanism

## Abstract

The formation and transformation of secondary iron (oxyhydr)oxides and their role in the stabilization of copper (Cu) and zinc (Zn) in acid mine drainage (AMD) after limestone treatment are worth studying to better understand the impacts of limestone AMD treatment. In this study, the wastewater from a copper mine ditch was sampled. Two different doses of limestone (S: 5.33 g L^−1^ and SS: 8.00 g L^−1^) were applied to adjust the pH range of the sampled AMD. The concentrations of Fe, Cu and Zn in the supernatant and the levels of iron (oxyhydr)oxides and heavy metals in AMD sediments were dynamically monitored for 300 days to analyze the transformation of the secondary iron mineral phase and the role iron (oxyhydr)oxides play in the removal and stabilization of Cu and Zn. The results showed that the pH rose rapidly to 6.82, decreased to 5.82 on the 150^th^ day, and finally decreased to approximately 4.63 by the 300^th^ day, when the dosage of limestone (S) was 5.33 g L^−1^. Goethite was the main form of iron oxides in the sediments. As the incubation time increased, so did the content of crystalline Fe (oxyhydr)oxides. In addition, the Cu and Zn content in the fraction of crystalline Fe (oxyhydr)oxides increased as the corresponding iron (oxyhydr)oxide increased. When the high dosage of limestone (8.00 g L^−1^ or SS) was applied, the pH remained at approximately at 7.46 during the whole period and goethite and lepidocrocite were present in the sediment. Amorphous/ poorly crystalline Fe-oxyhydroxide was the main product after SS limestone dosage, indicating that the risk of Cu and Zn reactivation in the sediment was higher with a higher limestone treatment dosage.

## Introduction

Acid mine drainage (AMD) is the water runoff produced from mineral mining activities. It is usually rich in iron, sulphate, and various toxic and harmful elements, and has a pH less than 5 (Romero et al., 2011). It is formed by a series of physicochemical reactions, such as dissolution, oxidation, and hydrolysis when sulfide is exposed to water or/and atmospheric oxygen during mining and the other related activities. AMD, if not properly handled, can seriously pollute rivers, lakes, groundwater, and soil and crops, causing ecological pollution and significant hazards to human health (Zhu et al., 2017). According to the United States (US) Forest Service, in 2010, more than 6,000 km of streams were polluted due to AMD from coal mines in the eastern US, and the cost of AMD treatment exceeded $10 billion (Naidu et al., 2019).

Limestone is a typical material used to treat AMD because of its mild alkalinity as well as being an abundant, easily accessible resource. Treatment and disposal methods of limestone should be adjusted based on whether active or passive neutralization is needed. Active neutralization is when the AMD is actively treated, in this case with a large amount of limestone, in order to quickly neutralize the pH and remove the heavy metals from the AMD. A process utilizing pulsed fluidized limestone beds was used to remediate acid mine drainage at the Friendship Hill National Historic Site in southwestern Pennsylvania. Over a fourteen-month period of time, 50 metric tons of limestone were used to treat 50 million liters of water. The influent water pH was 2.5 and the Al, Fe and Mn concentrations were 60, 200 and 10 mg L^−1^, respectively. After remediation, the effluent water pH ranged from 5.7 to 7.8 and 95% of the Al, 50 to 90% of the Fe, and <10% of the Mn were removed (Sibrell, Watten & Boone, 2003). Passive neutralization, which relies on natural processes to remediate AMD, is greatly affected by external factors such as influent water quality, terrain, and hydraulic conditions. The Anna S coal mine complex in Tioga County, Pennsylvania (USA) produced drainage with a pH of 2.8–3.6, containing 3–36 mg L^−1^ Al and 1–36 mg L^−1^ Fe. After remediation through parallel vertical flow ponds followed by aerobic wetlands, the AMD had a neutral pH, with less than 1 mg L^−1^ of Al and Fe (Hedin et al., 2010). Conversely, the Howe Bridge system, also in Pennsylvania, contains anoxic limestone drains (ALD) followed by successive alkalinity producing systems (SAPS). Initially, the pH of the AMD was 4.5 and the Fe concentration was 191 mg L^−1^. After remediation with this system, the effluent pH was still acidic (pH 5.5) and the Fe concentration was still high at 59 mg L^−1^ showing that this system not only increased the pH of the water, but also changed the iron content of the AMD, thereby affecting the migration and transformation of heavy metal ions after application of limestone to the AMD (Demchak, Morrow & Skousen, 2001).

Some research results show that the oxidation of Fe (II) and the hydrolysis of Fe (III) in AMD will form iron (hydroxyl)oxides precipitates, which can immobilize heavy metals by adsorption and co-precipitation and affect their migration and transformation in water under natural conditions (Bao, 2018). For example, the concentration of harmful elements such as Cu, Zn and As was greatly reduced downstream on the Clark Fork river due to the adsorption and co-precipitation of jarosite after a large amount of mining wastewater was added to the river (Hochella et al., 2005). In order to clarify the iron (oxyhydr)oxides responsible for the stabilization of copper and zinc under various pH conditions, many researchers have carried out relevant studies using a pure solution system (Cudennec & Lecerf, 2006). When the pH is acidic or alkaline, ferrihydrite will dissolve and recrystallize into goethite, but when the pH is neutral, hematite is formed. Without Pb (II), iron oxide in AMD forms goethite and magnetite, but with Pb, the iron oxide instead forms lepidocrocite (Liu et al., 2017). Heavy metal ions may also be re-released or co-precipitated during the transformation of iron (oxyhydr)oxides. Frierdich, Luo & Catalano (2011) found that in the process of Fe (II)-mediated transformation from Goethite to hematite, adsorbed Ni (II) would co-precipitate with dissolved Fe (III) into a hematite structure, while Zn (II) was released into a liquid phase.

Many factors affect AMD neutralization with limestone, so few researchers have studied the iron (oxyhydr)oxides responsible for the removal and stabilization of heavy metals in AMD after limestone treatment. For example, when a large dose of limestone is used for active treatment, iron-rich sludge is produced, and the stability of the heavy metals immobilized in the sludge is still unclear. Therefore, in this study, two doses of limestone were designed to adjust the pH of the AMD to 4.63−6.82 or approximately 7, respectively. After limestone treatment, the AMD supernatants and sediments were monitored dynamically for 300 days to explore (1) the changes of pH and heavy metal content in the supernatant between the two different limestone dose sizes; (2) the phase transformation of iron (oxyhydr)oxides and their role in the stabilization of Cu and Zn in AMD sediment under various pH conditions; and (3) the concentration and distribution of copper and zinc in the AMD sediment.

## Materials & Methods

### AMD sample and limestone

The AMD used in this study was taken from an open drainage ditch in the Dexing Copper Mine, Dexing, Jiangxi Province, China. The Dexing copper mine is the largest open-pit copper mine in Asia and its mining history dates back to 1958. The characteristics of the AMD are presented in Table 1.

Limestone (pH 9.32) was reagent which purchased from the Xilong Chemical Industry Incorporated Co., Ltd (Guangdong, China). Scanning electron microscope (SEM) images of limestone was presented in Fig. S1.

### Batch experiments

Limestone dosages were determined from the results of a short-term experiment done before the study began (Fig. S2). To evaluate the geochemical changes of Cu and Zn during the AMD neutralization process after limestone treatment, batch experiments were performed using 250 mL Erlenmeyer flasks wrapped with a transparent polyethylene film to prevent water evaporation. Each flask included 0.8 g (marked “S”) or 1.2 g (marked “SS”) of limestone and 150 mL of AMD. The flasks were shaken in a bed temperature incubator 8 hr/d at 120 vibration rpm at room temperature (25 °C). Two mixing treatments (S and SS) were both performed in triplicate.

A 10 ml sample of mixed solution was taken from the flask after 0.083 days (2 hr), 0.25 days (6 hr), 0.5 days (12 hr), 1 day, 3 days, 7 days, 14 days, 30 days, 60 days, 150 days and 300 days, centrifuged at 4,000 rpm for 10 min, and 8 ml of supernatant with 2% HNO_3_ was added. The solid phase precipitation was then cleaned with deionized water three times and then stored at 4 °C.

**Table 1:** Basic physical and chemical properties of the tested AMD.

pH	Fe	Cu	Zn	As	Cd	SO_4_^2−^
	mg L^−1^					
2.69	475.66 ± 11.42 (47.75% Fe^2+^)	85.88 ± 0.77	3.78 ± 0.53	–	0.027 ± 0.001	13070.41 ± 202.16

### Sequential extraction procedures of sediments

To evaluate the secondary iron oxides responsible for the Cu and Zn in the sediments, the sequential extraction sequence was set for the sediments treated with S or SS limestone dosage at room temperature on the 150^th^ day and the 300^th^ day. The sequential extraction methods used were based on the methods described by the literature (Dold, 2003; Kumpiene, Fitts & Mench, 2012; Tessier, Campbell & Bisson, 1979) and developed according to the results required for this experiment. The sequence of the steps is shown in Table 2. This study focused on iron-related extractants, so only steps 2, 3, and 4 are discussed in this paper. In addition, fractions 3 and 4 were merged into one fraction called crystalline Fe (oxyhydr)oxides. All extracts were filtered through 0.22 µm cellulose acetate syringe filters and stored at 4 °C prior to analysis using Flame Atomic Absorption Spectroscopy (FAAS).

**Table 2:** Adapted 4-step sequential extraction sequences developed for this study.

**Sequence**	**Extractant**	**L/S**	**washing step**	**Dissolved phases in this study**
(1) Exchangeable fraction	1M NH_4_-acetate(pH 4.5) shaken for 2 h at room temperature	25	10 mL deionized water	Gypsum, Metal Salts, Calcite
(2) Poorly crystalline Fe-oxyhydroxide fraction	0.2M NH_4_-oxalate(pH 3.0) shaken for 2 h in darkness at room temperature	25	NH_4_-oxalate Solution was used at L/S 12.5	Schwertmannite, lepidocrocite, Goethite, ferrihydrite
(3) Crystalline Fe (oxyhydr)oxide fraction	0.2M NH_4_-oxalate (pH 3.0) heated in water bath at 80 °C for 2 h	25	NH_4_-oxalate Solution was used at L/S 12.5	Higher Ordered Ferri -hydrite (*e.g.*, six-line), Goethite, Hematite
(4) Fe-Mn oxide fraction	0.04 M NH_2_OH-HCl in 25% (v/v) HO-acetate (pH 2) heated in water bath at 96 °C for 6 h	20	10 mL deionized water	Goethite Fe-Mn Oxides

### Chemical analysis and mineralogical determination

The water pH value was measured using a pH meter (PHS - 3C, Shanghai). To measure the metal contents, the AMD sample was filtered through a 0.22 µm filter, and the sediment was treated with a concentrated acid mixture of HCl - HNO_3_ –HF (2: 6: 2, v/v) using microwave digestion. Total Fe and Fe^2+^ in the solution were measured using the 1, 10-phenanthroline spectrophotometry method at 510 nm (UV-752, Shanghai), while the Cu and Zn contents were analyzed using FAAS (ContraAA-700; Analytik Jena, Jena, Germany).

The mineralogy of Fe (oxyhydr)oxides in the sediment was determined by powder X-ray diffraction (XRD, D8 ADVANCE Bruker, Germany), The XRD spectra used Cu K *α* radiation (40 kV, 40 mA) with 2 *θ* scanning from 10° to 70° in 0.02° increments and 0.1 s per step. The resulting spectra were interpreted using the “MDI JADE 5” software and “ICDD PDF (2004).” A scanning electron microscope (SEM, SU1510, Hitachi, Japan) was used to observe the surface morphology and microstructure. The FTIR spectra were recorded with a spectrophotometer (VERTEX 70; Bruker, Bremen, Germany), scanning from 4,000 to 400 cm^−1^ with a resolution of 4 cm^−1^ in KBr media (sample/KBr ratio of 1:100). The composition and oxidation state of the solid-phase elements were detected using X-ray photoelectron spectrometer techniques (XPS, Axis Ultra DLD; Kratos Analytical, Manchester, UK) with high-resolution spectra binding energies.

### Calculation and statistical analysis

The removal efficiency of metals in AMD solution could be calculated according to Eq. (1):

(1) \begin{eqnarray*}\text{metal}~\text{removal}~(\text{%})= \frac{{C}_{0}-{C}_{t}}{{C}_{0}} \times 100\text{%}\end{eqnarray*}

where C_0_ is the metal concentration in the original AMD solution (mg L^−1^), C_t_ is the metal concentration at time t in AMD solution (mg L^−1^).

Data were collected as previously described in Fisher (1956). Specifically, one-way analysis of variance (ANOVA) was performed on all the data using SPSS software (version 23.0). The data were presented as mean value ± standard error. Differences between means were determined using Duncan’s multiple range test (*P* < 0.05) (Duncan, 1955).

## Results and Discussion

### Variation of pH levels and metal content in AMD solutions

**Figure 1:**
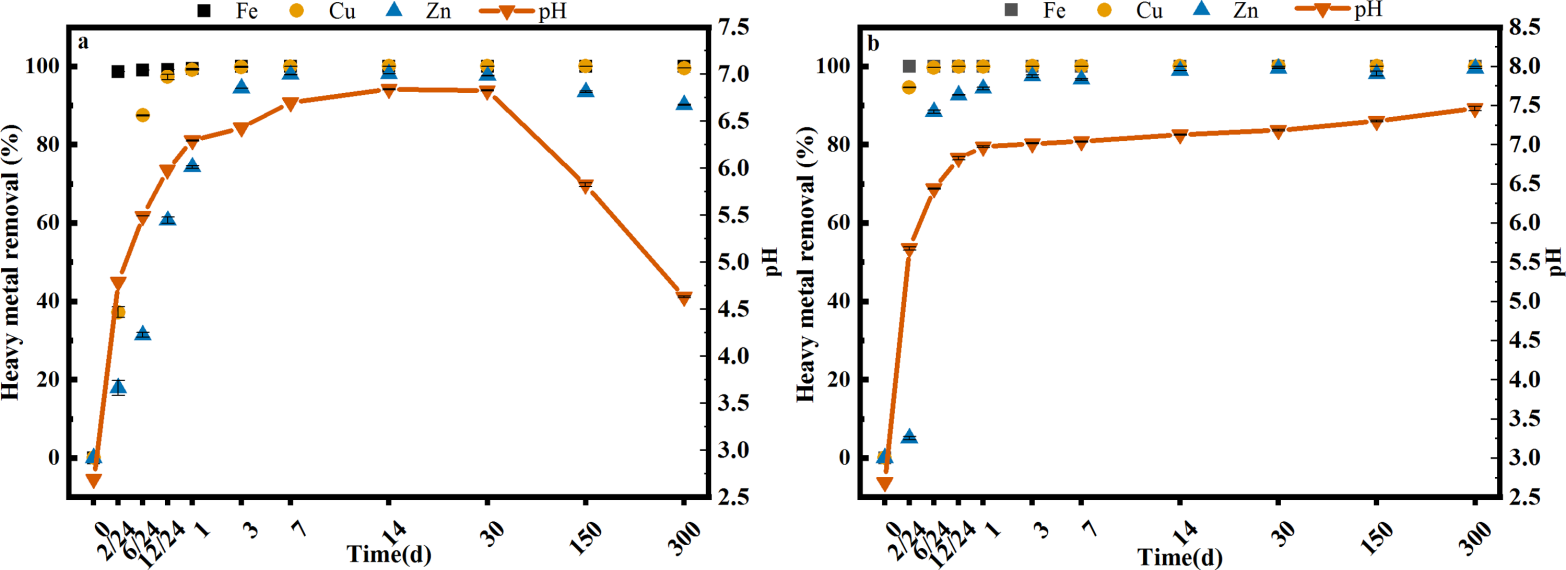
The change of pH and heavy metal content in the AMD with S (A) and SS (B) limestone addition at room temperature (25 °C). Error bars are standard error of the mean (*n* = 3).

Two different dosages of limestone (S and SS) were used to neutralize the AMD, and the results are shown in Fig. 1. In the first 30 days, the pH of the AMD increased rapidly and then settled around 6.82 (S) and 6.97 (SS), respectively, which was consistent with the pre-trial results (S1). Over a longer period of time, the pH of the AMD did not change significantly under the SS limestone treatment, but under the S limestone treatment, the pH continued to decrease to 5.82 after 150 days and 4.63 after 300 days. The secondary iron minerals in the sediments could continuously release H^+^ during phase transformation processes such as hydrolysis and re-precipitation after 30 days, which might explain the decreased pH after long-term batch treatment. The reaction mechanism can be explained by formulas 2 and 3 (Li et al., 2014; Liu et al., 2018):

(2) \begin{eqnarray*}M+F{\mathrm{e}}^{3+}+2{H}_{2}O\leftrightarrow MF\mathrm{e}OOH+3{H}^{+}~(M:metal)\end{eqnarray*}

(3) \begin{eqnarray*}MF{\mathrm{e}}_{8}{O}_{8}{ \left( OH \right) }_{8-2\mathrm{x}}{ \left( S{O}_{4}^{2-} \right) }_{\mathrm{x}}+2{\mathrm{xH}}_{2}O\rightarrow 8F\mathrm{e}OOH+\mathrm{x}S{O}_{4}^{2-}+M+2\mathrm{x}{H}^{+}.\end{eqnarray*}

Both limestone dosages showed significant iron removal rates with both removing 99% or more after just 6 h. The precipitation of iron also had significant effects on the co-precipitation of copper and zinc (Consani et al., 2019; Shi et al., 2021). Iron removal had a larger impact on copper than on zinc, removing 99.04% of copper after 1 day under both limestone dosages. Zinc removal was more variable, reaching 98.06% until day 14 and stabilizing to 90% (300 d) with the S limestone dosage, and greater than 99% using the SS limestone dosage. This showed that Cu and Zn could be effectively removed when the pH varied from 4.63 to 6.97, but that the precipitation and phase transformation of iron (oxyhydr)oxides might lead to the re-release of heavy metals.

By calculating the Q_SP_ of Zn and OH^−^ under experimental conditions, it was also discovered that the main removal mechanism of Zn was not hydroxide precipitation (Table S1). The removal of Cu and Zn ions in the AMD was also indirectly affected by coexisting ions and forms (such as Fe) in the AMD. Karthikeyan, Elliott & Cannon (1997) also confirmed that Cu tended to be removed through adsorption and coprecipitation with Fe (oxyhydr)oxides. Jonsson, Jonsson & Lovgren (2006) studied secondary iron mineral precipitation in AMD and showed that Zn was mainly removed through adsorption with lepidocrocite or other iron (oxyhydr)oxides. The removal rate of Zn was lower than the Cu removal rate in our experiments, which was also found in the research of Igarashi et al. (2020) on a two-step AMD neutralization ferrite-formation process with MgO and NaOH. Their research showed that the Cu had largely been removed after the first step when the pH of the AMD was 4.6, while the Zn was mainly present in the ferrite-formation sludge generated in the second step when the pH was 8.5. Zn also settled in the form of hydroxide precipitation and adsorption with crystalline iron (oxyhydr)oxides.

### Characterization of iron (oxyhydr)oxide phases in sediments

**Figure 2:**
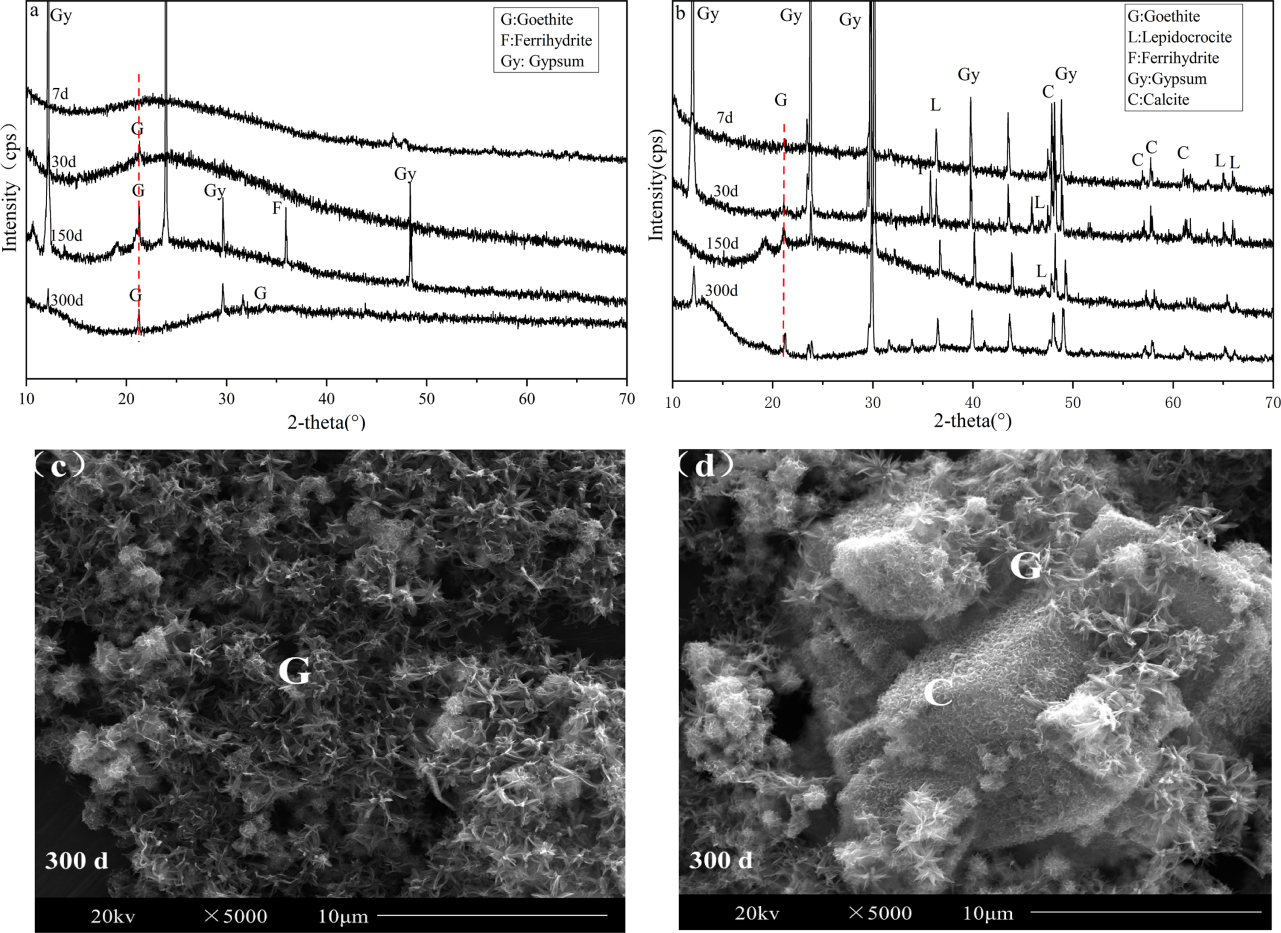
XRD pattern and SEM images of sediments of the AMD precipitates formed after S (A, C) and SS limestone (B, D) addition.

Our experimental results matched the results of previous studies that showed that the precipitation and phase transformation of iron might affect the changes of heavy metals in AMD. In order to understand the types, crystal styles and morphology of iron (oxyhydr)oxides, we analyzed the sediment using XRD and SEM (Fig. 2).

Iron existed mainly in an amorphous form when the limestone dosage was low (S treatment) before day 30. Goethite was first observed on day 30, and the characteristic peaks of it became more and more obvious as time passed. The ferrihydrite peak appeared on day 150, and the precipitation of ferrihydrite released H^+^, which caused the pH of the supernatant to decrease. This phenomenon was consistent with the results of previous studies. By day 300, the ferrihydrite peak disappeared, indicating that ferrihydrite might be transformed into goethite or weakened into amorphous form. Schwertmann & Murad (1983) reported the effect of pH on the transformation of ferrihydrite. The results showed that the final product of Fe oxide was goethite when the pH was 4 and the contents of Fe(OH)^2+^ and Fe(OH)^4−^ were the highest, which is more conducive to the formation of goethite. This also explains why goethite was the final iron mineral present after long batch experiments in our study when the pH of the AMD was 4.63 (Fig. 1). As illustrated in Fig. 2C, a large number of needle-like particles, which formed snowflakes, were relatively uniformly distributed in the entire viewing area, indicating that goethite with good crystallinity was formed (Liu et al., 2018; Rong et al., 2010).

Conversely, when the limestone dosage was high (SS treatment), goethite and lepidocrocite were observed on the 7^th^ day. Lepidocrocite is seldom found in natural AMD sediments with a low pH and high sulfate content, but can be found in alkaline conditions (Jonsson, Jonsson & Lovgren, 2006). Zhu et al. (2012) also showed that lepidocrocite, goethite, and ferrihydrite were dominant in AMD with neutral or alkaline conditions. Fan et al. (2023) also confirmed the formation of lepidocrocite and ferrihydrite in the CaCO_3_-FeCl_2_ system (pH = 7.5). The SEM images of the pure limestone (calcite) was shown in Fig. S1. Therefore, Fig. 2D showed that a small amount of acicular goethite was attached to the surface of the overdose calcite, indicating that most of the iron secondary minerals present were poorly crystalline minerals.

In summary, the iron formations were different under different pH conditions caused by differences in limestone dosages. Iron transformation could also counteract the pH variation and affect the stability of heavy metals in sediments.

### Composition of metals in sediment mineral phases

The precipitation and interaction between limestone and the phase transformation of Fe minerals played an important role in the stability of heavy metals in AMD sediments. The iron (oxyhydr)oxides responsible for copper and zinc stabilization in AMD was the focus of this study. Fourier Transform Infrared Spectroscopy (FTIR) provided information about the changes to iron (oxyhydr)oxides and heavy metal structures in the sediment as batch time extended to 300 days.

**Figure 3:**
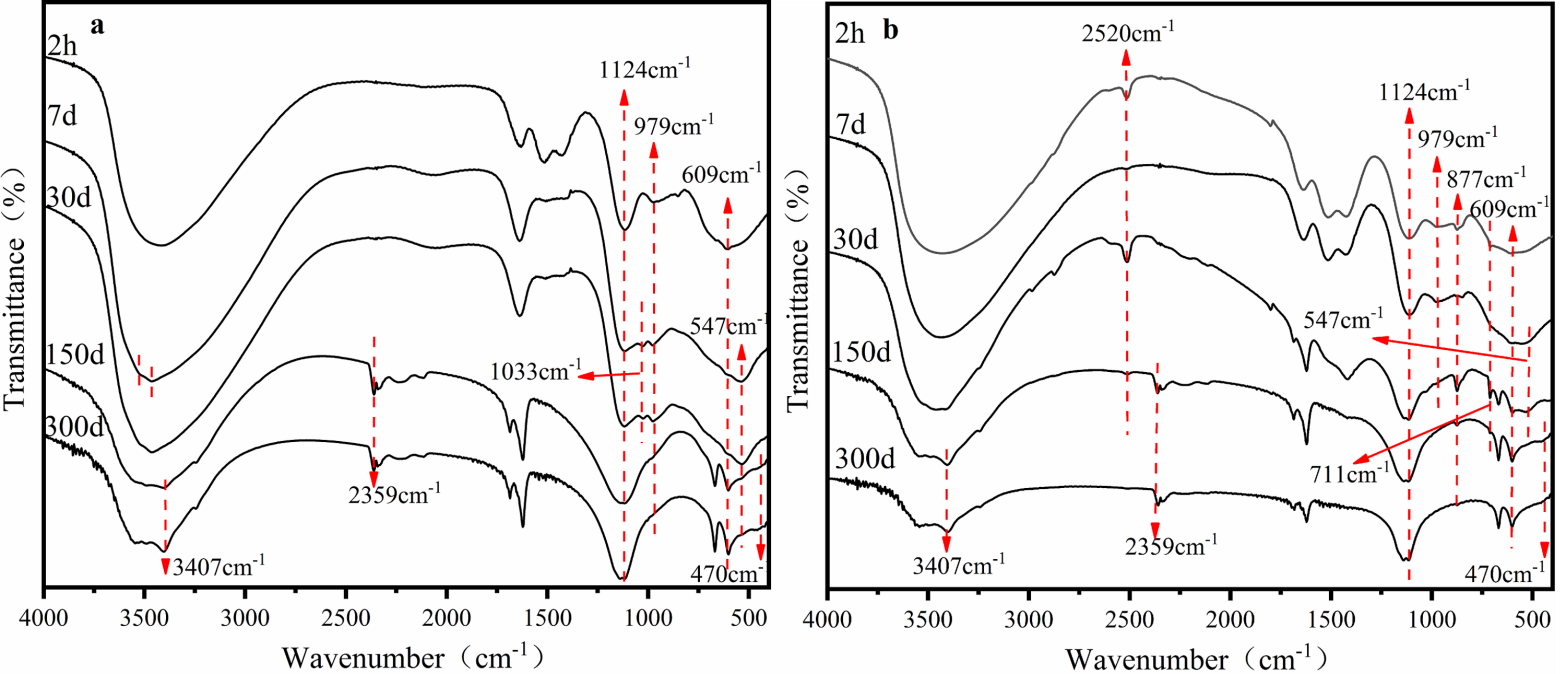
FTIR pattern of sediments of the AMD precipitates formed after S (A) and SS (B) limestone addition.

The FTIR characterization of sediments under the S limestone dosage is shown in Fig. 3A. The wide peak from 3,200 cm^−1^ to 3,700 cm^−1^ was the characteristic peak of hydroxyl. There was a small difference between the surface hydroxyl groups and the hydrated hydroxyl groups: the former were 3,400 cm^−1^ and the latter were approximately 3,200 cm^−1^ due to differences in H-bonding (Antelo et al., 2012; Kubicki, Paul & Sparks, 2008; Vener, Shenderovich & Rykounov, 2013). Figure 3 shows that the hydroxyl groups in the sediments were mainly surface hydroxyl groups. On the 7^th^ day, the wide peak at 3,400 cm^−1^ gradually divided into two peaks, and the characteristic peak of hydrated hydroxyl appeared at the same time because of the large number of heavy metals in the sediment, such as Cu and Zn. The formation of -O-Cu and -O-Zn destroyed the structure of hydroxyl, resulting in the unstable splitting of groups into peaks (Kubicki, Paul & Sparks, 2008). The stable stretching vibration absorption peak of -OH at 3,407 cm^−1^ appeared on the 150^th^ day, which was the characteristic peak of the Fe hydroxyl oxide of goethite (Gagliano et al., 2004; Lewis, 1986). On the 30^th^ day, the peak at 547 cm^−1^ attributed to Cu replaced H in Fe-O-H to form Fe-O-Cu (Wang et al., 2011). Both the characteristic peak and the bending vibration absorption peak at 1,033 cm^−1^ of Fe-OH gradually disappeared with time. This may be because the amorphous Fe-oxyhydroxide transformed to a crystalline phase. A new peak of Fe-O at 470 cm^−1^ and a characteristic peak at 2,359 cm^−1^ appeared on the 150^th^ day. Gan et al. (2017) found in the co-precipitation experiment of Fe and heavy metals that the peak at 2,359 cm^−1^ was the characteristic peak of the combination of heavy metals with iron (oxyhydr)oxides. This was consistent with our conclusion.

The FTIR characterization of sediments with the addition of the SS limestone dosage is shown in Fig. 3B. The absorption peak at 2,520 cm^−1^ and 711 cm^−1^ corresponded to the antisymmetric and symmetric stretching vibration peak and in-plane bending vibration of CO_3_^2−^, respectively. The bending vibration absorption peak of Fe-OH at 877 cm^−1^ was observed after 2 h. The absorption peak gradually weakened with time because lepidocrocite was gradually transforming into other mineral phases. At the same time, the absorption peak of Fe-O at 470 cm^−1^ appeared. The characteristic peak of the combination of heavy metals with iron (oxyhydr)oxides at 2,359 cm^−1^ also formed at the early stage of the reaction and increased with time. The stretching vibration absorption peak of goethite-OH at 3,407 cm^−1^ appeared on day 30.

The results showed that the Fe mineral phase in the AMD was constantly changing under both limestone dosages, but there were stable characteristic peaks of goethite in the end, which was also consistent with the XRD results. At the same time, part of the heavy metals combined with the Fe-oxyhydroxide and surface hydroxyl groups of the sediment.

### Fate and speciation of copper and zinc in sediments

**Figure 4:**
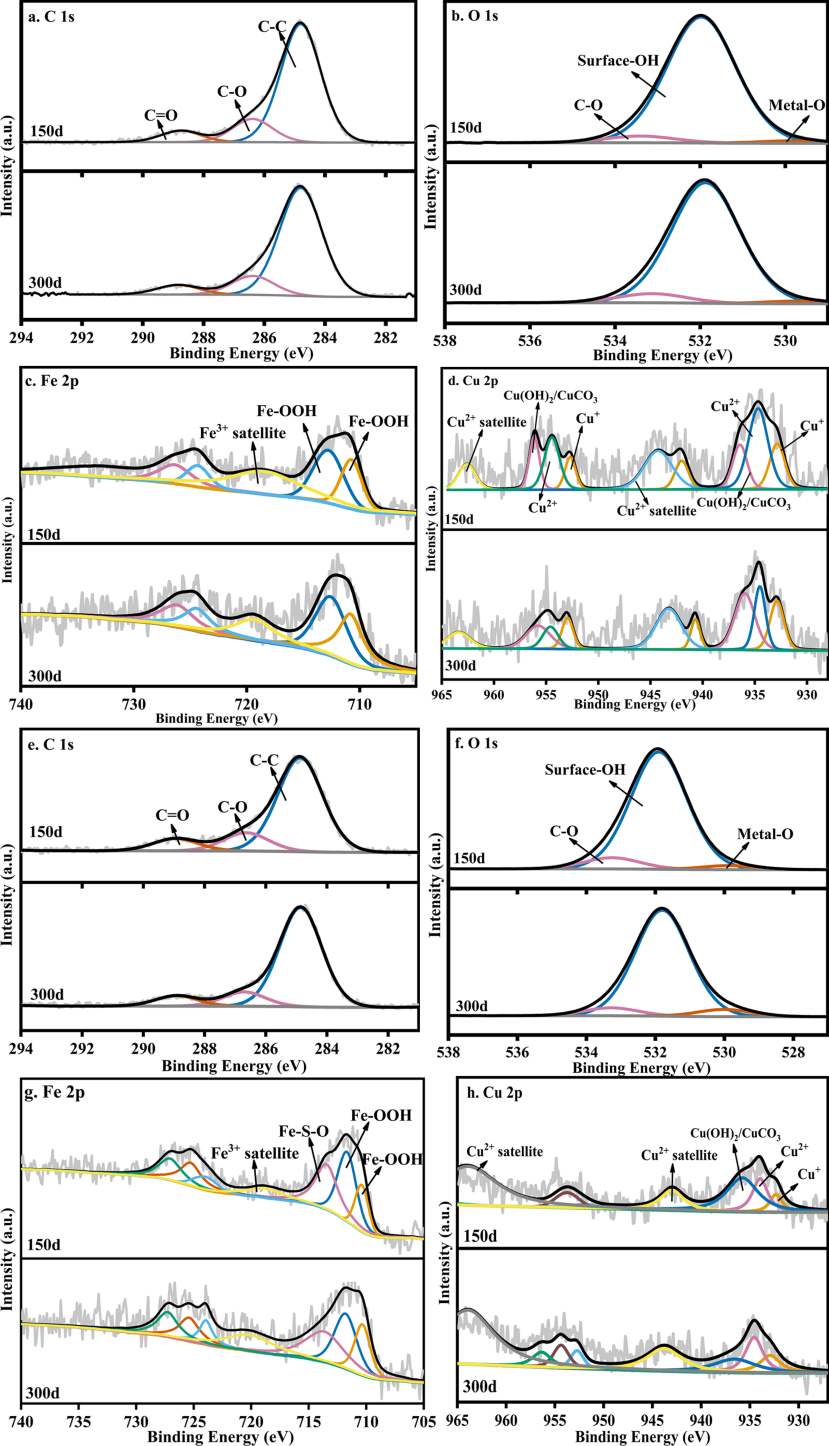
XPS pattern of sediments on the 150th day and the 300th day: S limestone dosage (A–D) and SS limestone dosage (E–H).

In order to further analyze the long-term stabilization mechanism of the heavy metals in the sediments, the sediments on the 150^th^ day and the 300^th^ day were characterized by XPS. Because the initial Zn content was much lower than that of Cu and Fe in the water samples, only the Fe 2p and Cu 2p peaks were fitted to the sediment to explore the relationship between the changes of iron secondary minerals and heavy metals, as shown in Fig. 4.

Figure 4B shows the O 1s core level spectra of the sediments, where the binding energy (BE) positions at 532 eV and 529–530 eV corresponded to surface-OH and Metal-O, respectively (Ardizzone & Bianchi, 1999). The area of surface-OH at 532 eV decreased from 93.22% to 91.05% over time, while the area of Metal-O at 530 eV increased from 1.85% to 2.03%, indicating that the heavy metals bound to surface-OH groups decreased and the heavy metals bound to O increased. The fitting result of Fe 2p (Fig. 4C) showed that the two divided peaks at 711.8 eV and 712.4 eV corresponded to Fe-OOH, which also confirmed the conclusion (Section 3.2) that the Fe (oxyhydr)oxide was ferrihydrite or goethite in the sediment. The fitting results of Cu 2p are shown in Fig. 4D. The peak around 934.0 eV could be regarded as Cu^2+^ with an octahedral structure because Cu^2+^ replaced Fe in octahedral structure, and Fe (oxyhydr)oxides such as goethite and lepidocrocite contained the octahedral structure of Fe (Crane & Sapsford, 2018). The peak around 932.5 eV was Cu^+^, which could be regarded as the characteristic peak of Cu_2_S or Cu_2_O (Suponik, Winiarski & Szade, 2015). The peak at 935.5 eV indicated the presence of Cu(OH)_2_, CuCO_3,_ or Cu_2_(OH)_2_CuCO_3_ in the sediment (Yan, Sun & Meng, 2018). Cu^2+^ decreased over time, indicating that the copper complexes were released when the Fe (oxyhydr)oxide changed from poorly crystalline ferrihydrite to goethite. The increase of Cu^+^ and Cu(OH)_2_/CuCO_3_ also indicated that the released Cu continued to be fixed in the sediment in the form of Cu_2_S/Cu_2_O or combined with hydroxyl and carbonate.

However, Cu mainly existed in the form of Cu(OH)_2_/CuCO_3_ (Fig. 4H) on the 150^th^ day under the SS limestone dosage. The peak area of Cu(OH)_2_/CuCO_3_ decreased significantly and the peak area of Cu^2+^ increased significantly, indicating that most Cu precipitated in the form of Cu(OH)_2_/CuCO_3_ at first, then bonded to the carbonate while the surface hydroxyl group entered the crystal structure of Fe (oxyhydr)oxide, so it could remain stable in the sediments on the 300^th^ day.

**Figure 5:**
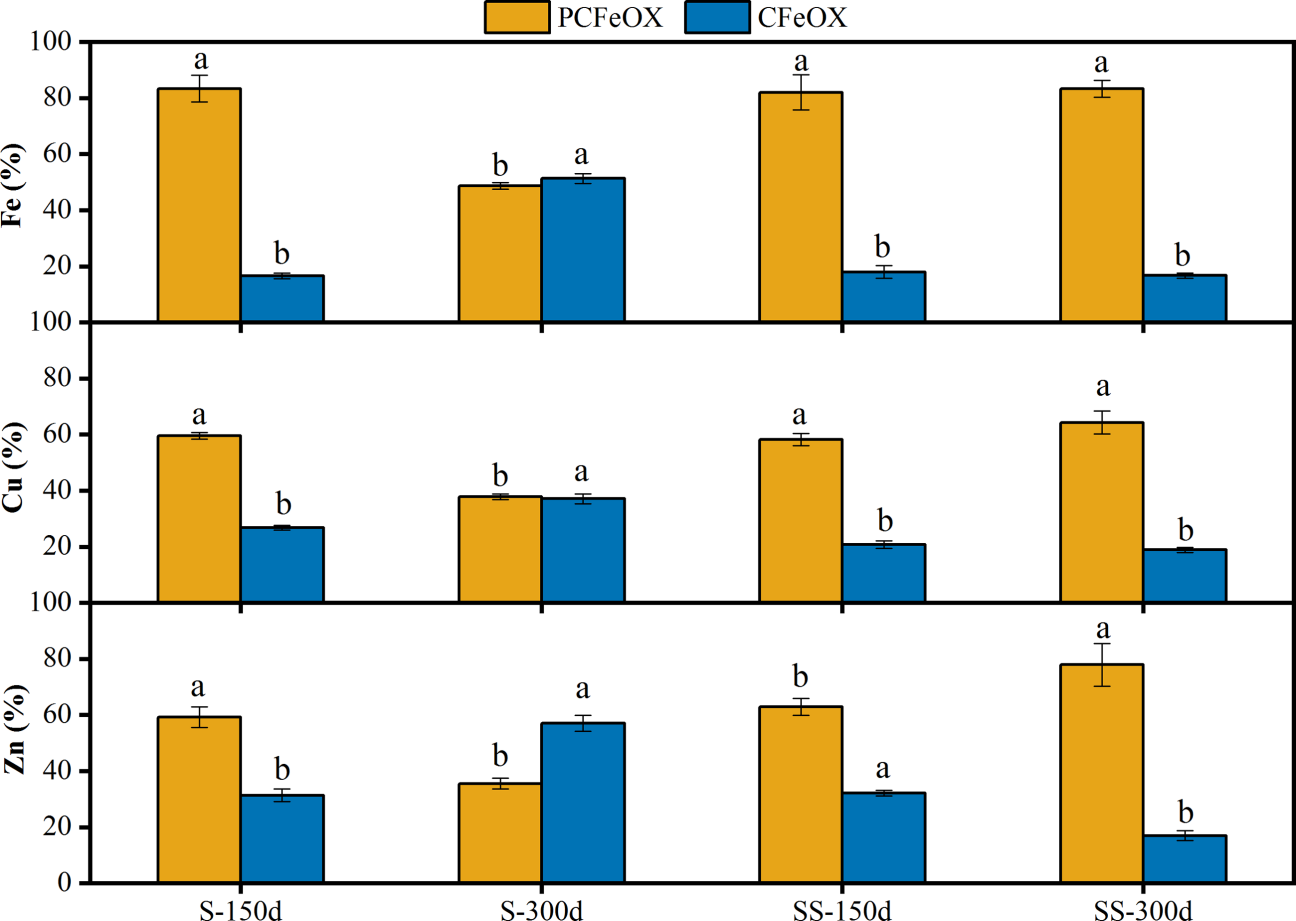
Fractional distribution of Fe, Cu and Zn in sediments of AMD precipitates after the S and SS limestone dosages at room temperature (25 °C). PCFeOX, poorly crystalline Fe-oxyhydroxides, CFeOX, crystalline Fe (oxyhydr)oxides. Different letters indicated significant differences at different incubation time in same S/SS limestone addition and PCFeOX/ CFeOX bonded fraction (*P* < 0.05). Error bars are standard error of the means (*n* = 3).

In order to further explore the combination characteristics of Cu and Zn with poorly crystalline/crystalline Fe (oxyhydr)oxides in the sediments, the content of AMD sediments under the two dosages of limestone were analyzed on the 150^th^ day and 300^th^ day. The results are shown in Fig. 5.

As shown in Fig. 5, the pH decreased from 5.82 to 4.63, but the content of crystalline Fe (oxyhydr)oxides increased from 16.63% to 51.33% with the S limestone dosage between day 150 and day 300. Combined with the experimental results of XRD, the results show that the poorly crystalline ferrihydrite was transformed into crystalline goethite, indicating that low pH conditions were more beneficial to the transformation of poorly crystalline Fe-oxyhydroxides into crystalline forms. Under the SS limestone dosage, Fe mainly existed in the form of poorly crystalline Fe-oxyhydroxides, and there was no obvious change over time. Her (2007) also showed that the re-crystallization transformation process of Fe (oxyhydr)oxides was very slow when the pH was 7–8. The Fe was mainly goethite and lepidocrocite in the sediments, with goethite existing primarily in a poorly crystalline form.

Under the S limestone dosage, the fraction of Cu bound to crystalline Fe (oxyhydr)oxide increased significantly from 26.77% to 37.12%, and the fraction of Cu bound to poorly crystalline Fe-oxyhydroxides decreased from 59.54% to 37.79%. These results, combined with the above XPS results, show that in the process of Fe oxide transformation from poorly crystalline to crystalline, Cu bounding to crystalline Fe (oxyhydr)oxide was released. A total of 47.59% of the Cu content was released, combining with the newly formed crystalline Fe (oxyhydr)oxide, with the rest binding to carbonate/hydroxyl and Cu^2+^. Under the SS limestone treatment, the fraction of Cu bound to poorly crystalline Fe-oxyhydroxides increased from 58.21% to 64.34%, while the fraction of Cu bound to crystalline Fe (oxyhydr)oxides decreased from 20.75% to 18.87%. This shows that under the SS limestone dosage, the Fe (oxyhydr)oxides remained in poorly crystalline form for a long time in the sediments, and complexed other forms of copper ions over time. This was consistent with the conclusion of the XPS analysis.

Due to the low Zn content and unclear XPS characterization, the fixation mechanism of Zn in the sediments could only be inferred through speciation analysis. Under the S limestone dosage, the fraction of Zn bound to crystalline Fe (oxyhydr)oxides increased by 25.69% with the increase of time and crystalline Fe (oxyhydr)oxide. As the pH decreased, Zn was re-released into the solution, but the part that bound to Fe (oxyhydr)oxides still increased by 1.94%. This shows that Zn was not only removed by the surface adsorption of crystalline Fe (oxyhydr)oxides, but also embedded into its lattice during the phase transformation from poorly crystalline to crystalline Fe (oxyhydr)oxides. Under the SS limestone dosage, the fraction of Zn bound to poorly crystalline Fe-oxyhydroxides increased by 14.96%, and the fraction of Zn bound to crystalline Fe (oxyhydr)oxides decreased by 15.08%. There was no obvious change in the part combined with Fe. Due to the continuous rise of liquid pH, Zn remained stable in the sediment, but the increased Zn content in crystalline Fe (oxyhydr)oxides led to the re-release of Zn. Therefore, attention should be paid to the risk of secondary pollution when reusing the sediment.

## Conclusions

Our study exhibited that the effect of limestone addition on the pH of AMD depended on both the limestone dosage and the incubation time, which also determined the size and crystal style of iron (oxyhydr)oxides. When the limestone dosage was 5.33 g L^−1^, the pH was stable at approximately 6.82 from day 7 to day 30, but decreased to 4.63 by day 300. Under this dosage, iron (oxyhydr) oxides existed mainly as goethite in AMD sediments by day 300. The proportion of crystaline Fe (oxyhydr)oxides size increased from 16.63% to 51.33%, and Cu and Zn bonded increased by 10.35% and 25.69%, respectively, from day 150 to day 300. However, when the limestone dosage was 8.00 g L^−1^, the pH continuously increased to 7.46 by day 300. Under this dosage, poorly crystal/amorphous iron (oxyhydr)oxides, such as lepidocrocite and goethite were the most common forms by day 300. Also, heavy metals mainly retained in the poorly crystalline Fe-oxyhydroxides bound fraction. Cu and Zn increased by 6.13% and 14.96%, respectively, from day 150 to day 300. Our findings indicated that the risk of Cu and Zn reactivation in the sediments varied with time under different limestone dosages.

##  Supplemental Information

10.7717/peerj.14663/supp-1 Supplemental Information 1 SEM image of the pure limestone

10.7717/peerj.14663/supp-2 Supplemental Information 2 Changes of pH in AMD with different limestone addition

10.7717/peerj.14663/supp-3 Supplemental Information 3 Calculation of Q_sp of zinc hydroxide in AMD under SS limestone addition

10.7717/peerj.14663/supp-4 Supplemental Information 4 Concentration and removal rate of Fe, Cu and Zn in supernatant

10.7717/peerj.14663/supp-5 Supplemental Information 5 Fractional distribution of Fe, Cu and Zn in sediments

10.7717/peerj.14663/supp-6 Supplemental Information 6 Basic physical and chemical properties of the tested AMD

10.7717/peerj.14663/supp-7 Supplemental Information 7 Changes of pH in AMD with different limestone addition

10.7717/peerj.14663/supp-8 Supplemental Information 8 Calculation of Qsp of zinc hydroxide in AMD under SS limestone addition

## Additional Information and Declarations

### Data Availability

The following information was supplied regarding data availability: The raw measurements are available in the Supplemental Files.

## References

[ref-1] Antelo J, Fiol S, Gondar D, Lopez R, Arce F (2012). Comparison of arsenate, chromate and molybdate binding on schwertmannite: surface adsorption vs anion-exchange. Journal of Colloid and Interface Science.

[ref-2] Ardizzone S, Bianchi CL (1999). Electrochemical features of zirconia polymorphs. The interplay between structure and surface OH species. Journal of Electroanalytical Chemistry.

[ref-3] Bao YP (2018). Diversity of iron- and sulfur-cycling microorganism and the role of microbial activity in Fe(III) hydroxysulfate mineral transformations in a river affected by acid mine drainage. PhD thesis.

[ref-4] Consani S, Ianni MC, Dinelli E, Capello M, Cutroneo L, Carbone C (2019). Assessment of metal distribution in different Fe precipitates related to acid mine drainage through two sequential extraction procedures. Journal of Geochemical Exploration.

[ref-5] Crane RA, Sapsford DJ (2018). Selective formation of copper nanoparticles from acid mine drainage using nanoscale zerovalent iron particles. Journal of Hazardous Materials.

[ref-6] Cudennec Y, Lecerf A (2006). The transformation of ferrihydrite into goethite or hematite, revisited. Journal of Solid State Chemistry.

[ref-7] Demchak J, Morrow T, Skousen J (2001). Treatment of acid mine drainage by four vertical flow wetlands in Pennsylvania. Geochemistry: Exploration, Environment, Analysis.

[ref-8] Dold B (2003). Speciation of the most soluble phases in a sequential extraction procedure adapted for geochemical studies of copper sulfide mine waste. Journal of Geochemical Exploration.

[ref-9] Duncan DB (1955). Multiple range and multiple F tests. Biometrics.

[ref-10] Fan Q, Wang L, Fu Y, Wang Z (2023). Impacts of coexisting mineral on crystallinity and stability of Fe(II) oxidation products: implications for neutralization treatment of acid mine drainage. Journal of Hazardous Materials.

[ref-11] Fisher RA (1956). Statistical methods and scientific inference.

[ref-12] Frierdich AJ, Luo Y, Catalano JG (2011). Trace element cycling through iron oxide minerals during redox-driven dynamic recrystallization. Geology.

[ref-13] Gagliano WB, Brill MR, Bigham JM, Jones FS, Traina SJ (2004). Chemistry and mineralogy of ochreous sediments in a constructed mine drainage wetland1 1Associate editor: P. A. Maurice. Geochimica et Cosmochimica Acta.

[ref-14] Gan M, Li MM, Zeng J, Liu XX, Zhu JY, Hu YH, Qiu GZ (2017). Acidithiobacillus ferrooxidans enhanced heavy metals immobilization efficiency in acidic aqueous system through bio-mediated coprecipitation. Transactions of Nonferrous Metals Society of China.

[ref-15] Hedin R, Weaver T, Wolfe N, Weaver K (2010). Passive treatment of acidic coal mine drainage: the Anna S mine passive treatment complex. Mine Water and the Environment.

[ref-16] Her N (2007). Transformation of 2-line ferrihydrite and its effect on arsenic adsorption. D. Phil. Thesis.

[ref-17] Hochella MF, Moore JN, Putnis CV, Putnis A, Kasama T, Eberl DD (2005). Direct observation of heavy metal-mineral association from the clark fork river superfund complex: implications for metal transport and bioavailability. Geochimica et Cosmochimica Acta.

[ref-18] Igarashi T, Herrera PS, Uchiyama H, Miyamae H, Iyatomi N, Hashimoto K, Tabelin CB (2020). The two-step neutralization ferrite-formation process for sustainable acid mine drainage treatment: removal of copper. Science of the Total Environment.

[ref-19] Jonsson J, Jonsson J, Lovgren L (2006). Precipitation of secondary Fe(III) minerals from acid mine drainage. Applied Geochemistry.

[ref-20] Karthikeyan KG, Elliott HA, Cannon FS (1997). Adsorption and coprecipitation of copper with the hydrous oxides of iron and aluminum. Environmental Science and Technology.

[ref-21] Kubicki JD, Paul KW, Sparks DL (2008). Periodic density functional theory calculations of bulk and the (010) surface of goethite. Geochemical Transactions.

[ref-22] Kumpiene J, Fitts JP, Mench M (2012). Arsenic fractionation in mine spoils 10 years after aided phytostabilization. Environmental Pollution.

[ref-23] Lewis DG (1986). Infrared absorption of surface hydroxyl groups and lattice vibrations in lepidocrocite (*γ*-FeOOH) and Boehmite (*γ*-AlOOH). Clay Minerals.

[ref-24] Li J, Kawashima N, Fan R, Schumann RC, Gerson AR, Smart RS (2014). Method for distinctive estimation of stored acidity forms in acid mine wastes. Environmental Science & Technology.

[ref-25] Liu CS, Li FB, Chen MJ, Liao CZ, Tong H, Hua J (2017). Adsorption and stabilization of lead during Fe(II)-catalyzed phase transformation of ferrihydrite. Acta Chimica Sinica.

[ref-26] Liu QY, Chen BH, Haderlein S, Gopalakrishnan G, Zhou YZ (2018). Characteristics and environmental response of secondary minerals in AMD from dabaoshan mine. Ecotoxicology and Environmental Safety.

[ref-27] Naidu G, Ryu S, Thiruvenkatachari R, Choi Y, Jeong S, Vigneswaran S (2019). A critical review on remediation, reuse, and resource recovery from acid mine drainage. Environmental Pollution.

[ref-28] Romero FM, Nunez L, Gutierrez ME, Armienta MA, Ceniceros-Gomez AE (2011). Evaluation of the potential of indigenous calcareous shale for neutralization and removal of arsenic and heavy metals from acid mine drainage in the Taxco Mining Area, Mexico. archives of Environmental Contamination and Toxicology.

[ref-29] Rong X, Chen W, Huang Q, Cai P, Liang W (2010). Pseudomonas putida adhesion to goethite: studied by equilibrium adsorption, SEM, FTIR and ITC. Colloids and Surfaces B: Biointerfaces.

[ref-30] Schwertmann U, Murad E (1983). Effect of pH on the formation of goethite and hematite from ferrihydrite. Clays and Clay Minerals.

[ref-31] Shi M, Min X, Ke Y, Lin Z, Yang Z, Wang S, Peng N, Yan X, Luo S, Wu J, Wei Y (2021). Recent progress in understanding the mechanism of heavy metals retention by iron (oxyhydr)oxides. Science of the Total Environment.

[ref-32] Sibrell PL, Watten B, Boone T (2003). Remediation of acid mine drainage at the friendship hill national historic site with a pulsed limestone bed process. Electrometallurgy and Environmental Hydrometallurgy.

[ref-33] Suponik T, Winiarski A, Szade J (2015). Species formed on iron surface during removal of copper ions from aqueous solutions. Physicochemical Problems of Mineral Processing.

[ref-34] Tessier A, Campbell P, Bisson M (1979). Sequential extraction procedure for the speciation of particulate trace metals. Analytical Chemistry.

[ref-35] Vener MV, Shenderovich IG, Rykounov AA (2013). A qualitative study of the effect of a counterion and polar environment on the structure and spectroscopic signatures of a hydrated hydroxyl anion. Theoretical Chemistry Accounts.

[ref-36] Wang S, Wang N, Li CL, Zhang JJ, Dou S (2011). FTIR spectroscopic analysis of Cu2+ adsorption on hematite and bayerite. Spectroscopy and Spectral Analysis.

[ref-37] Yan XD, Sun JL, Meng YA (2018). Experimental insight into the chemical corrosion mechanism of copper with an oil-in-water emulsion solution. RSC Advances.

[ref-38] Zhu J, Zhang P, Yuan SH, Liao P, Qian A, Liu XX, Tong M, Li LN (2017). Production of Hydroxyl radicals from oxygenation of simulated AMD due to CaCO3-induced pH increase. Water Research.

[ref-39] Zhu MQ, Legg B, Zhang HZ, Gilbert B, Ren Y, Banfield JF, Waychunas GA (2012). Early stage formation of iron oxyhydroxides during neutralization of simulated acid mine drainage solutions. Environmental Science & Technology.

