# Supplementary figures and images for: Iron (oxyhydr)oxides are responsible for the stabilization of Cu and Zn in AMD after treatment with limestone
Source: PeerJ. 2023 Jan 30;11:e14663. doi: 10.7717/peerj.14663 (PMC9897064; doi:10.7717/peerj.14663)

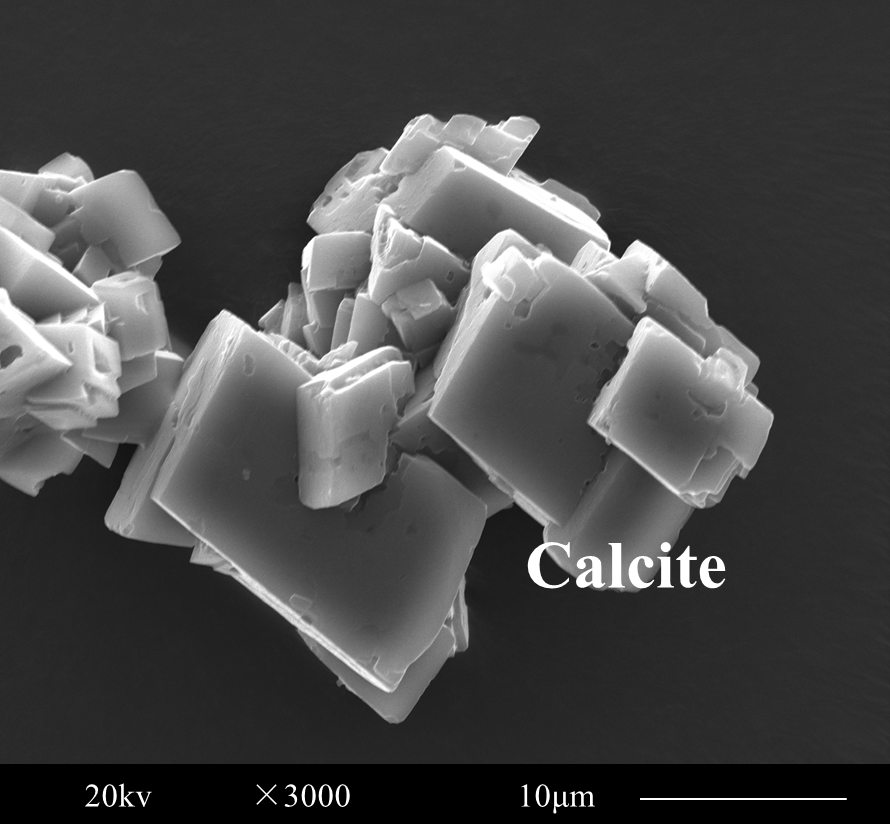

Supplement: Supplemental Information 1 [file peerj-11-14663-s001.png]

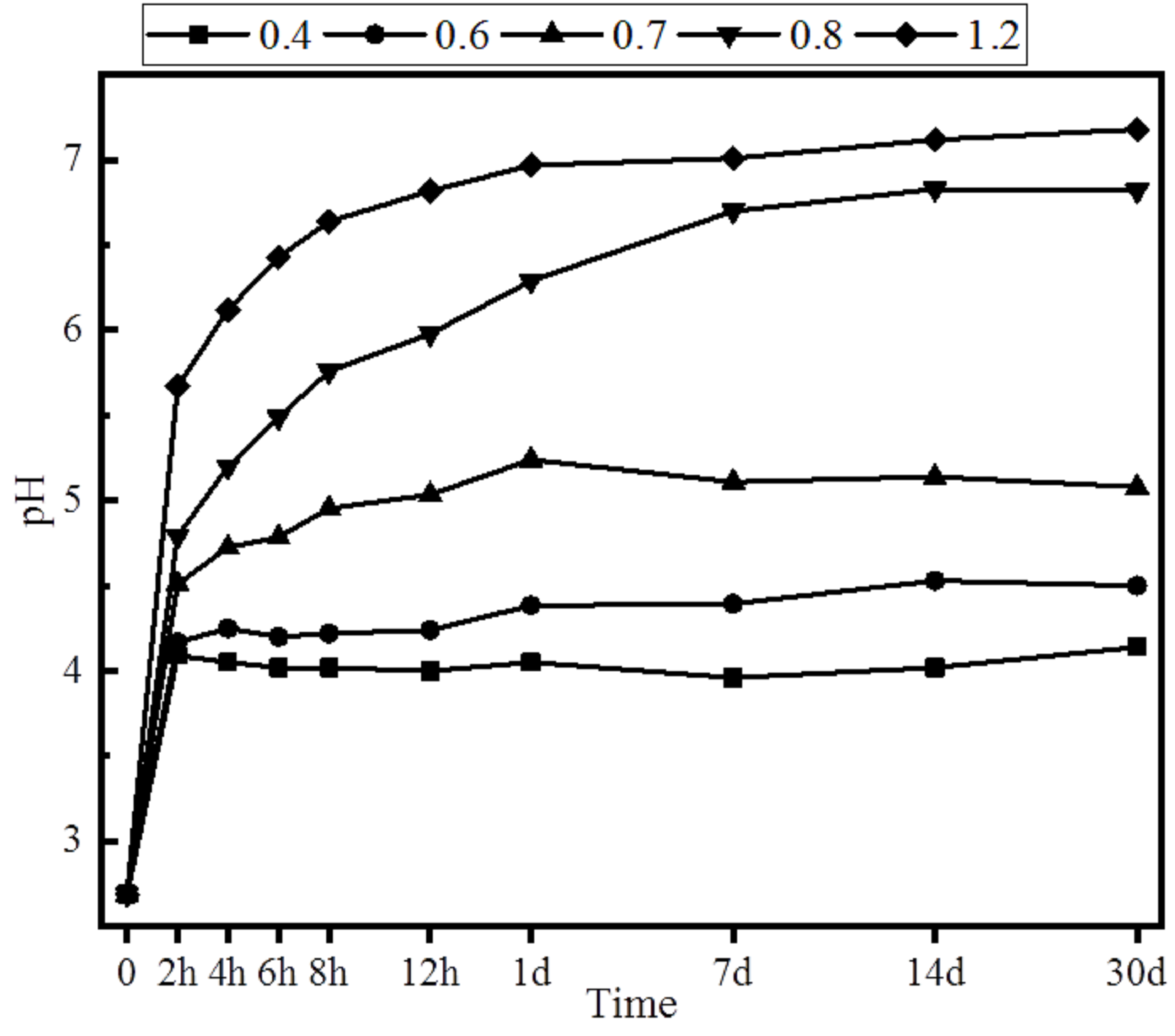

Supplement: Supplemental Information 2 [file peerj-11-14663-s002.png]
