# Supplementary material for: Iron (oxyhydr)oxides are responsible for the stabilization of Cu and Zn in AMD after treatment with limestone
Source: PeerJ. 2023 Jan 30;11:e14663. doi: 10.7717/peerj.14663 (PMC9897064; doi:10.7717/peerj.14663)
Supplement: Supplemental Information 3 [file peerj-11-14663-s003.docx]

**Table S2.** Calculation of Q_sp_ of zinc hydroxide in AMD under SS limestone addition

| Time | pOH | Zn(mol L^-1^) | [Zn^2+^][OH^-^]^2^ | Ksp |
| --- | --- | --- | --- | --- |
| 2h | 8.33 | 5.49*10^-5^ | 1.20*10^-21^ | 1.2*10^-17^ |
| 6h | 7.56 | 6.70*10^-6^ | 5.08*10^-21^ |  |
| 12h | 7.17 | 4.26*10^-6^ | 1.95*10^-20^ |  |
| 24h | 7.03 | 3.28*10^-6^ | 2.86*10^-20^ |  |
| 1d | 6.99 | 1.48*10^-6^ | 1.55*10^-20^ |  |
| 7d | 6.96 | 1.96*10^-6^ | 2.36*10^-20^ |  |
| 14d | 6.87 | 6.58*10^-7^ | 1.20*10^-20^ |  |
| 30d | 6.82 | 3.40*10^-7^ | 7.79*10^-21^ |  |
| 150d | 6.70 | 1.11*10^-6^ | 4.42*10^-20^ |  |
| 300d | 6.54 | 3.31*10^-7^ | 2.75*10^-20^ |  |
